# A Paradigm Shift in Progress: Generative AI’s Evolving Role in Mental Health Care

**DOI:** 10.2196/82369

**Published:** 2025-12-17

**Authors:** John Torous, Andrea Cipriani

**Affiliations:** 1 Division of Digital Psychiatry, Beth Israel Deaconess Medical Center, Harvard Medical School, 20 Overland St, Suite 202Boston, MA, 02215, United States, 1 6176676700; 2 Department of Psychiatry, University of Oxford, Oxford, United Kingdom; 3 Oxford Health NHS Foundation Trust, Warneford Hospital, Oxford, United Kingdom; 4 Oxford Precision Psychiatry Lab, Oxford Health Biomedical Research Centre, Oxford, United Kingdom

**Keywords:** AI, artificial intelligence, LLM, mental health, apps, large language model

## Abstract

Generative artificial intelligence (AI) is reshaping mental health but the direction of that change remains unclear. In this commentary, we examine the recent evidence and trends in mental health AI to identify where AI can provide value for the field while avoiding the pitfalls that have challenged the smartphone app and VR space. While AI technology will continue to improve, those advances alone are not enough to move AI from mental wellness to psychiatric tools and a new generation of clinical investigation, integration, and leadership will unlock the full value of AI.

The role of generative AI technology in mental health has rapidly evolved from conceptual potential to emerging real-world implementation. In a 2018 review of AI chatbots for mental health, only 10 studies were identified [1]; today, the literature includes hundreds of studies. This substantial growth in research parallels reports of millions of individuals using these tools for emotional support, as well as an increasing interest among clinicians to understand their utility better [2]. Although the mental health field will not transform overnight, the evidence suggests that a paradigm shift is already underway.

Rapid advances in the technical aspects of large language models make it difficult to predict the future of mental health AI. The ability of these models to have lucid conversations has dramatically improved in the last 12 months and is likely to continue to advance in the next 12 months. However, lessons from past technology trends, such as mental health apps and virtual reality, provide insights into where the field is likely headed, as forecasted in Figure 1 below. This article explores why mental health AI is poised for continued growth, while also identifying areas where current interest may be misplaced, undervalued, or underexplored. While AI may offer potential cost savings, building and running AI systems is costly, and those savings will only materialize if AI addresses priorities where it can create value. By adopting a thoughtful strategy that prioritizes real-world potential, organizations, developers, clinicians, and patients should position themselves as leaders in shaping the next generation of mental health care and generate that value.

**Figure 1.**
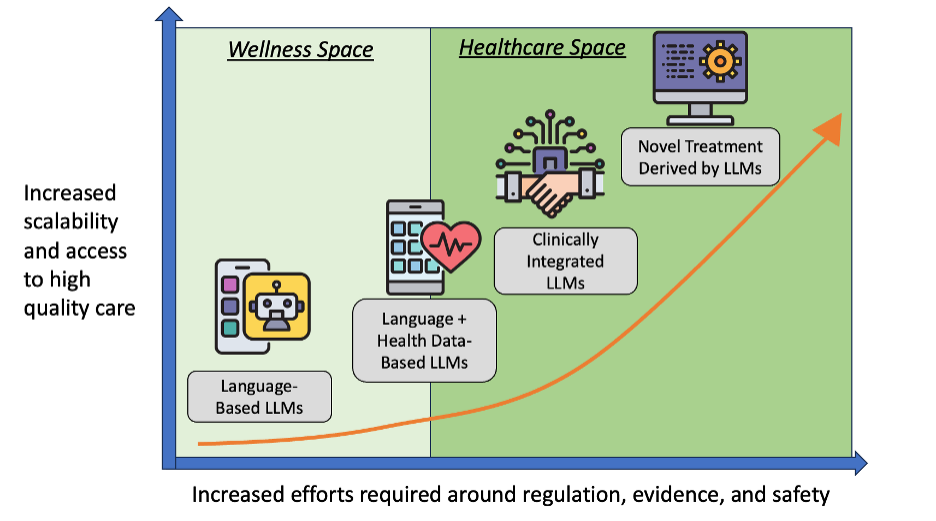
Today, the growth of LLMs (large language models) in health care, especially mental health, remains in the wellness space but with access to new health care data and later clinical integration, it will rapidly move into the health care space. As the field moves towards LLM-based treatment delivery, the need for regulation and evidence will increase.

AI has already succeeded in one key area where prior digital mental health technologies have lagged. The real-world adoption of previous mental health technologies, such as apps and VR, by patients and clinicians has been limited [3]. Mental health apps have gained notoriety for low rates of patient engagement, and clinicians’ uptake of these apps has been equally disappointing [4,5]. One of the largest clinical implementations of mental health apps within a large health care system reached over one-third of a million patients, but reported low clinician adoption and patient engagement, limiting the impact of large-scale rollouts [6]. In contrast, AI chatbots appear to be tools that many patients may already be turning to [7], even without these chatbots being marketed, designed, or trained to offer emotional support. Likewise, many clinicians are allowing AI agents into clinical sessions via AI scribes, creating an opportunity no other health technology, apart from computerized medical records, has ever had. While there is still much we do not know about patient engagement and clinician use of AI, early results suggest a notably different trajectory compared to apps and VR. Hospitals, electronic medical records, and big technology companies are all working to create easier and more powerful ways to use AI in health care in an integrated fashion, making it soon hard not to use it.

However, early adoption headwinds, which favor AI, are alone insufficient to drive further health care adoption (and a material change in clinical practice). In summer 2025, one of the first mental health AI chatbots announced that it would no longer support its flagship product, citing regulatory challenges as a key factor [8]. The regulatory landscape remains in flux, and in the United States, rhetoric has shifted from a conservative ‘do no harm’ to a recent suggestion that the government should encourage a ‘try it first approach.’ [9] How this rhetoric may or may not translate into policy will undoubtedly impact the pace of adoption. The current fragmentation of mental health AI policy at an individual state level in the United States (and also in other countries more globally) presents a less visible but critical challenge for widespread health care use [10]. Without a clear standard for evaluating the risks and benefits of AI tools in mental health, benchmarking what successful use of AI looks like will pose a barrier to clinical, regulatory, and ethical evaluation. AI companies are aware of this, and some are already proposing their own benchmarks for measuring success [11], but will also need to transparently share standard metrics and outcomes data for the field to gain trust. Meanwhile, medical societies and clinical teams are likely to follow suit and present alternative evaluation metrics soon, with the need to define harm and adverse events most pressing. To identify meaningful outcomes and validated processes, there is a need for a methodologically sound and evidence-based approach. Carrying out co-designed living systematic reviews allows for the collection and assessment of all the relevant evidence from different types of data (from randomized trials to observational studies) in a continuously updated manner [12]. While there is an ambition in clinical settings for individuals and their caregivers to be at the center of shared decision-making, research processes have often excluded people with lived experience and have not always focused on the outcomes that matter most to them. Subjective experiences of illness, treatment, and recovery can offer insights that neither research nor clinical expertise alone can fully capture [13]. These insights can challenge assumptions, highlight blind spots. and inform more meaningful approaches to care. This approach has been used in many areas of mental health to develop recommendations for future research and inform the prioritization process [14,15]. Integrating the perspectives of those with lived experience within existing research structures and decision-making practices requires a methodological reorientation in psychiatry. Generative AI is an ideal field for extending this innovative way of synthesizing evidence.

Currently, the excitement and overenthusiasm surrounding AI in mental health, from both academic and industry partners, can distract from understanding the current state of the field and its potential. For example, on the academic side, a recent randomized controlled trial of an AI therapy chatbot drew considerable attention even though the control group was a waitlist control [16]. The challenge is that in academic research, almost anything is superior to a waitlist control, and thus, such a study can establish feasibility but not efficacy. Indeed, the editor of the journal in which the waitlist control paper was published was quoted in response to being asked about the impact of this paper: “Perhaps we are not at a GPT4.0 moment but more like a GPT 1.0 (circa 2018) moment, [17].” Likewise, on the industry side, Microsoft recently announced its newest AI models were four times superior to clinicians, forgetting to highlight that the clinician control group was not permitted to use the internet or consult with colleagues [18]. Yet, even if a particular AI therapy chatbot proved only as effective as a digital placebo (perhaps a chatbot that discusses the weather) and the Microsoft AI model was only 40% as effective as a clinician, each of these outcomes would still be very impressive and exciting. These more modest outcomes do not negate the paradigm shift but help contextualize that it will not happen next month, as often feels the case when reading various headlines without the full details. Assessing outcomes in light of their actual science and rigor offers the benefit of highlighting the open questions in the field: *“Are AI chatbots effective at therapy or is there something therapeutic for some people in just talking to any chatbot regardless of whether it offers therapy or not,”* and *“how can clinicians and AI work together to be more effective?”*

The current focus on AI chatbots to deliver mental health therapy has garnered notable attention and warrants a deeper consideration of the challenges and work that remains to be done. There is no doubt that AI chatbots are superior at language [19], and that language is a core component of effective talking-therapy–based treatments. However, it is unknown how well these AI chatbots can deliver therapy, especially to patients with more severe mental health illnesses. Even with generative AI, as of August 2025, no chatbot is willing to assume medical or legal responsibility for therapy in patients with a mental illness. One company, noting that it created a new AI model specifically for mental health and therapy, today informs users in crisis that they are not allowed to use the service [20]. Perhaps current models of therapy, which the current AI chatbots are trained on, are not the ideal ones for chatbots to deliver. There may remain a new paradigm of psychotherapy to be uncovered. Therapies like CBT, the most popular among chatbots, were developed in a different era, and there is no reason new or alternative therapies cannot be developed for the unique world of generative AI.

For AI to play a role as a health tool and eventually deliver therapy, we must acknowledge that it may also pose risks and find ways to mitigate those risks. While there remains considerable and justified attention to errors that AI can make [21], such errors are also to be expected. It is not reasonable to expect AI agents not trained for mental health care to answer every single question or case correctly. It is doubtful that any clinician today is perfect, either. However, it is likely that mental health AI programs will continue to learn, and the rates of errors will become lower. For example, the latest data on ChatGPT 5, run against the HealthBench benchmark for health care use cases, showed significant improvements over all prior versions. However, without knowing how these AIs perform or what their training data is, there is concern that their scores may be more due to pattern matching than actual intelligence [22]. Closed, proprietary models thus pose additional risks. Health care systems will likely need more resources and support to run the infrastructure to securely deploy LLMs. But we also need to focus on risks that are intrinsic and cannot be fixed with more resources. While not well documented in the medical literature, popular press outlets have reported several cases of AI chatbot users developing psychotic symptoms [23]. While it is likely that many of these users harbored preexisting risks for psychosis, today we do not know and need to explore what this emerging phenomenon represents [24]. Likewise, there is growing concern that some users may form parasocial relationships with AI chatbots [25], which leads to adverse mental health outcomes, especially when the AI is removed, updates, or refuses to engage further in certain discussions. Issues of dependence and addiction, with some chatbot AI users forming their own internet support forums after not being taken seriously by the medical community [26], warrant serious attention.

Yet talking therapies, even those guided by AI, are only useful if they reach the right person at the right time. But given the well-known challenges around the reliability and accuracy of mental health diagnosis, there is a parallel need for innovation in how we define these conditions. While it is easy to criticize the DSM (Diagnostic and Statistical Manual of Mental Disorders), competing models such as HiTOP (Hierarchical Taxonomy of Psychopathology) and RDOC (Research Domain Criteria) have had limited impact on care as they cannot easily guide treatment decisions, like when or what type of treatment a particular patient needs [27]. Mental health AI may finally enable the field to reconceptualize mental illness and consider new definitions and categories through enabling a new generation of measurement. Beyond words and language, AI agents can already capture images to perform facial emotional analysis and voice to conduct personality and emotional assessments with a surprisingly high degree of accuracy [28]. They can also ingest mobile and digital phenotyping data, such as steps, geolocation, and sleep, and use this information to guide more accurate and personalized clinical predictions [29]. Such a new conceptualization of mental illness will not result in immediate new treatment, but moving beyond the current categorical nosologies to more personalized and dynamic clusters based on multimodal data will itself be a material change for biological research, drug discovery, prevention, and targeted treatment. By creating new theories of what mental illnesses are, drawing on this next generation of evidence-based and measurement-based care, we will not need AI chatbots to use older therapies to treat outdated versions of illness. We they will also need them to help guide novel prediction and delivery of new treatments, informed by human-AI interaction design, for the prevention of reconceptualized mental illnesses.

AI will transform mental health, but like all paradigm shifts, the transformation will not be linear or straightforward. Already, many commercial aspects of AI, such as clinician-facing scribes, are becoming commodities that are given away for free. As more aspects of AI become commodities, the value will shift towards their clinical validation and implementation. And as regulation, privacy, and ethics take on a larger role in the space, further shifts will occur. Hopefully, these trends will accelerate the development of safe and effective AI for mental health, and what may be perceived as delays are actually rapid progress of a paradigm shift in action. When considering the actual risks and benefits of AI beyond the current hype, it is clear that the field of psychiatry can and will continue to have a leadership role in shaping the next generation of research, care, diagnosis, and prevention.

## Abbreviations

AI: artificial intelligence
LLM: large language model
